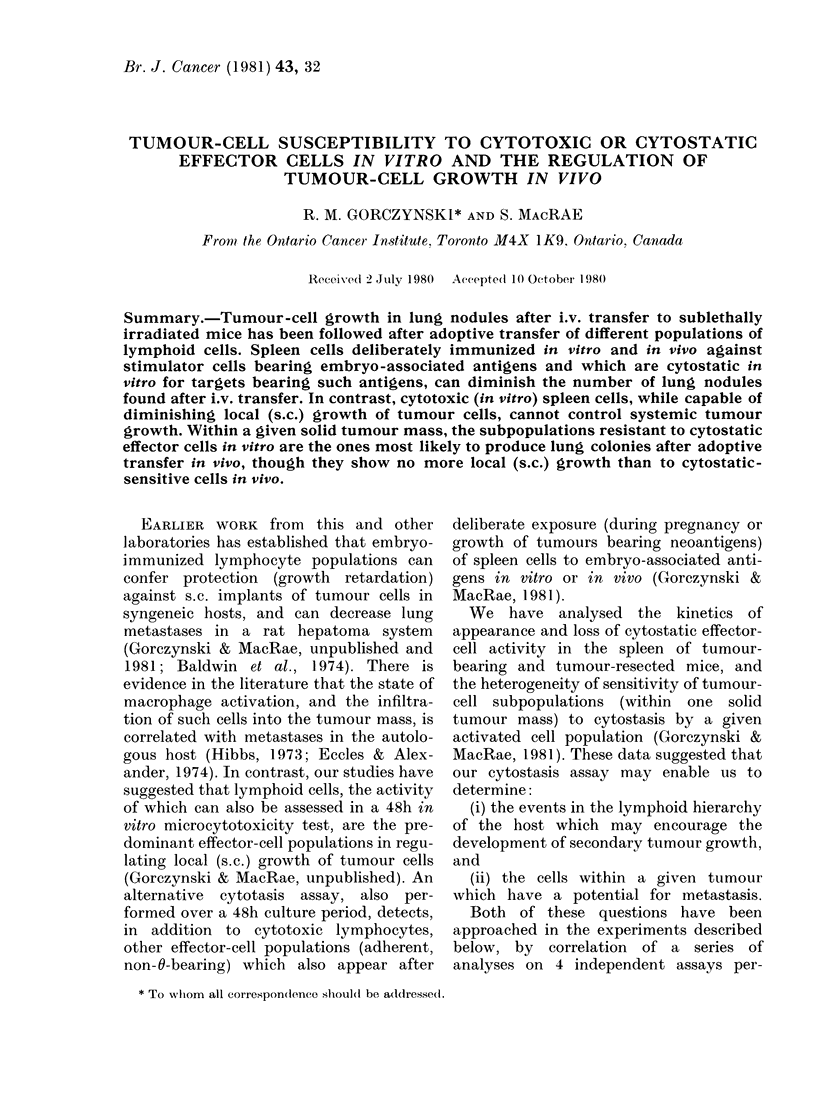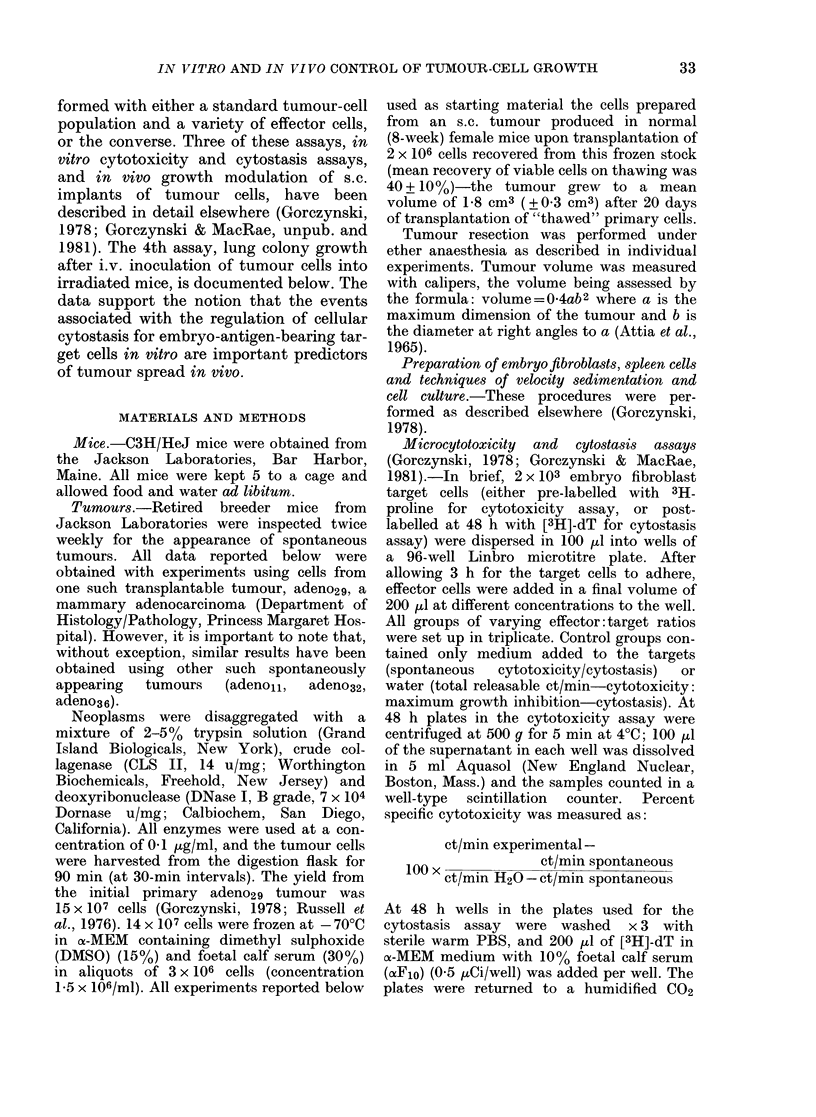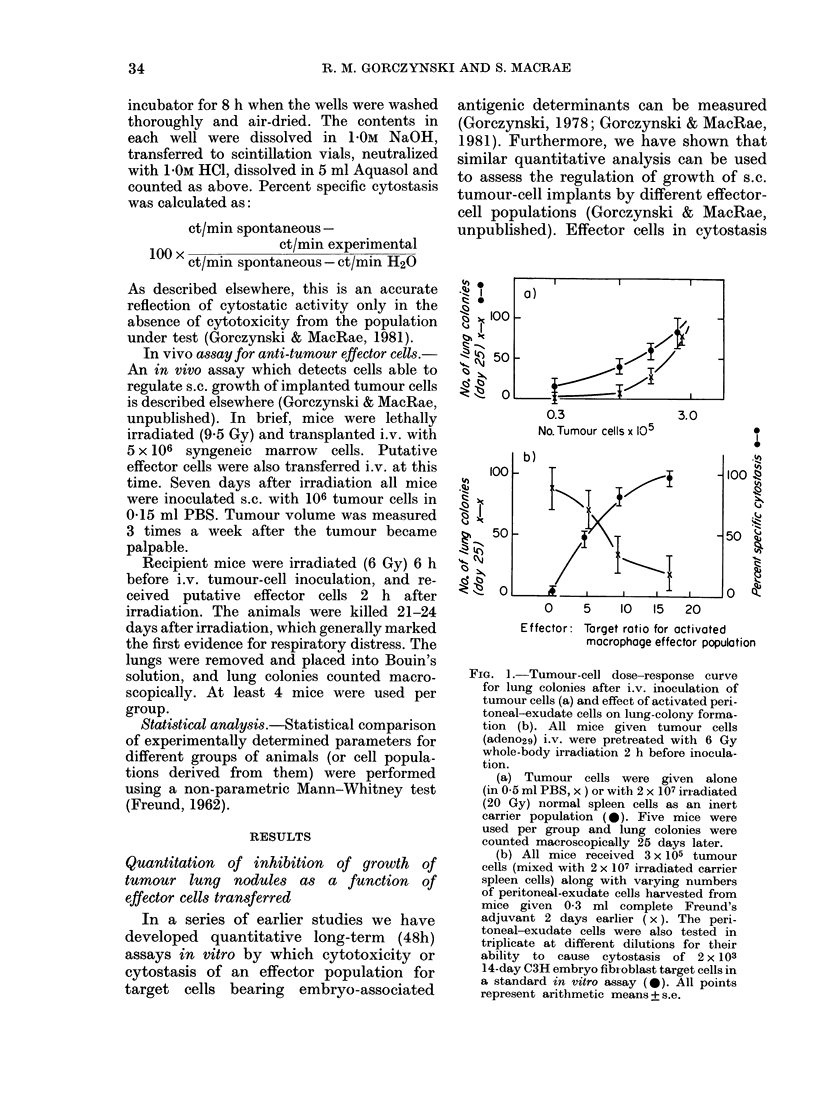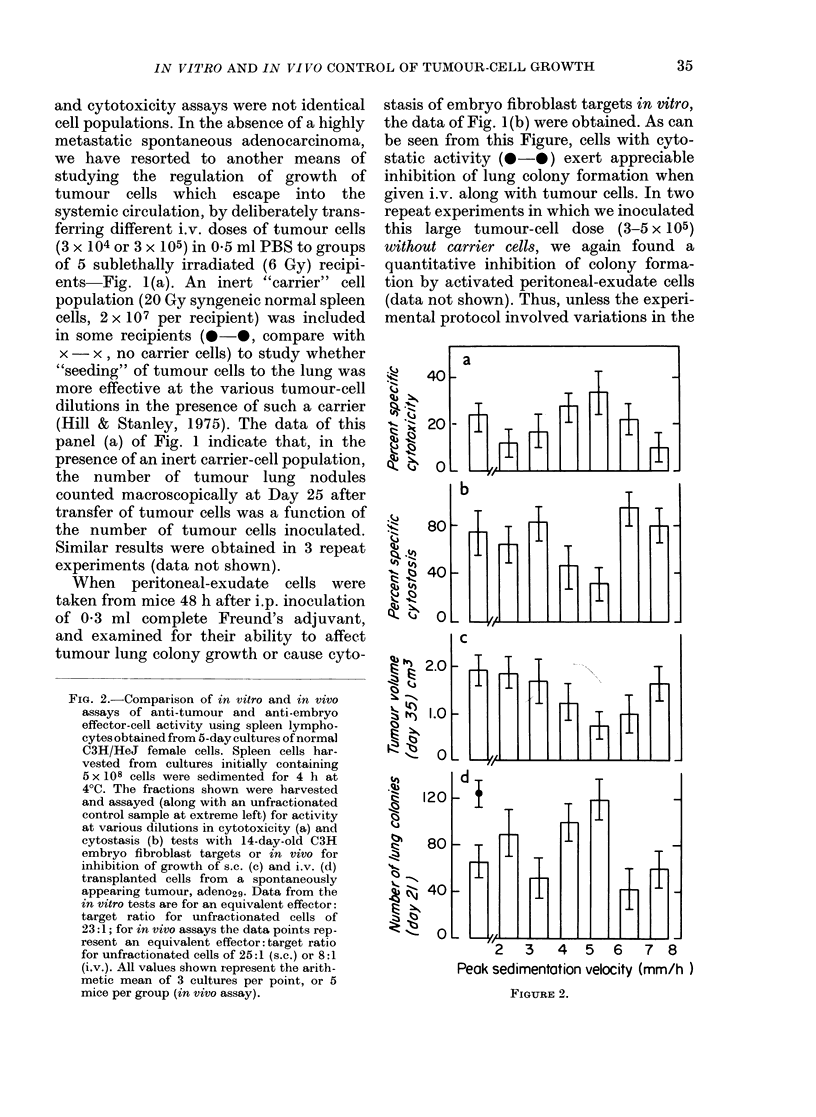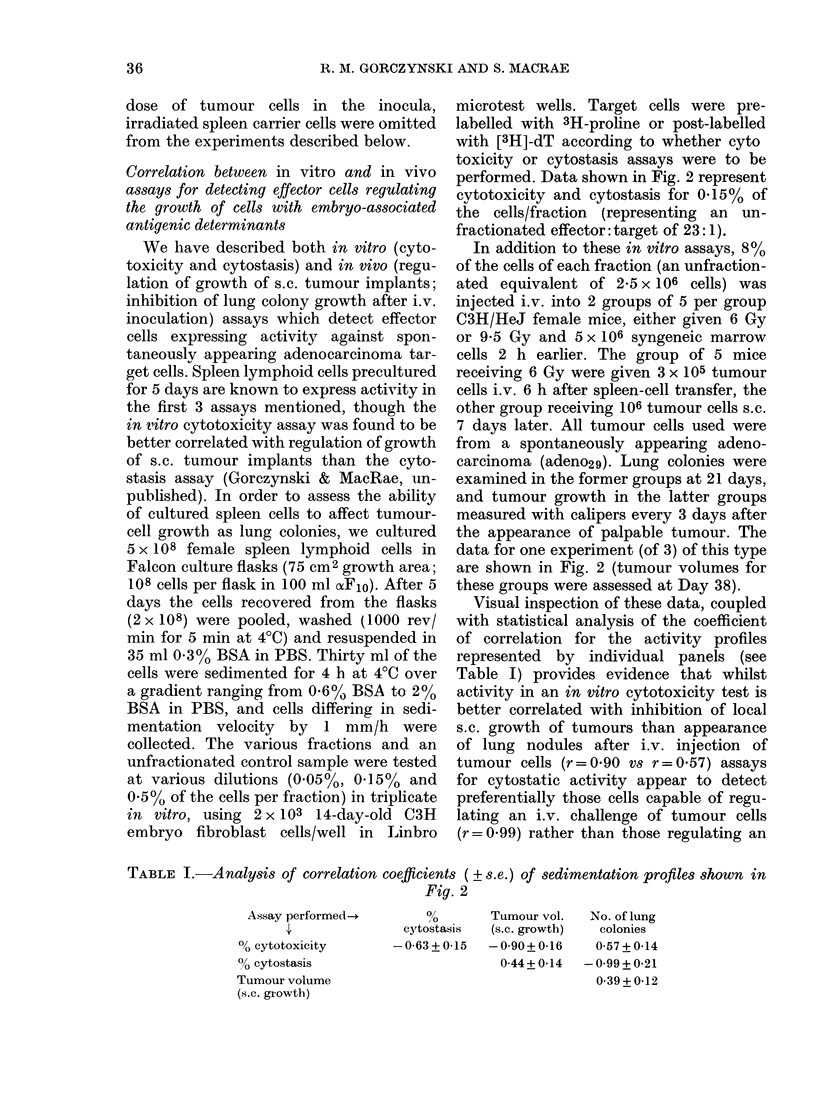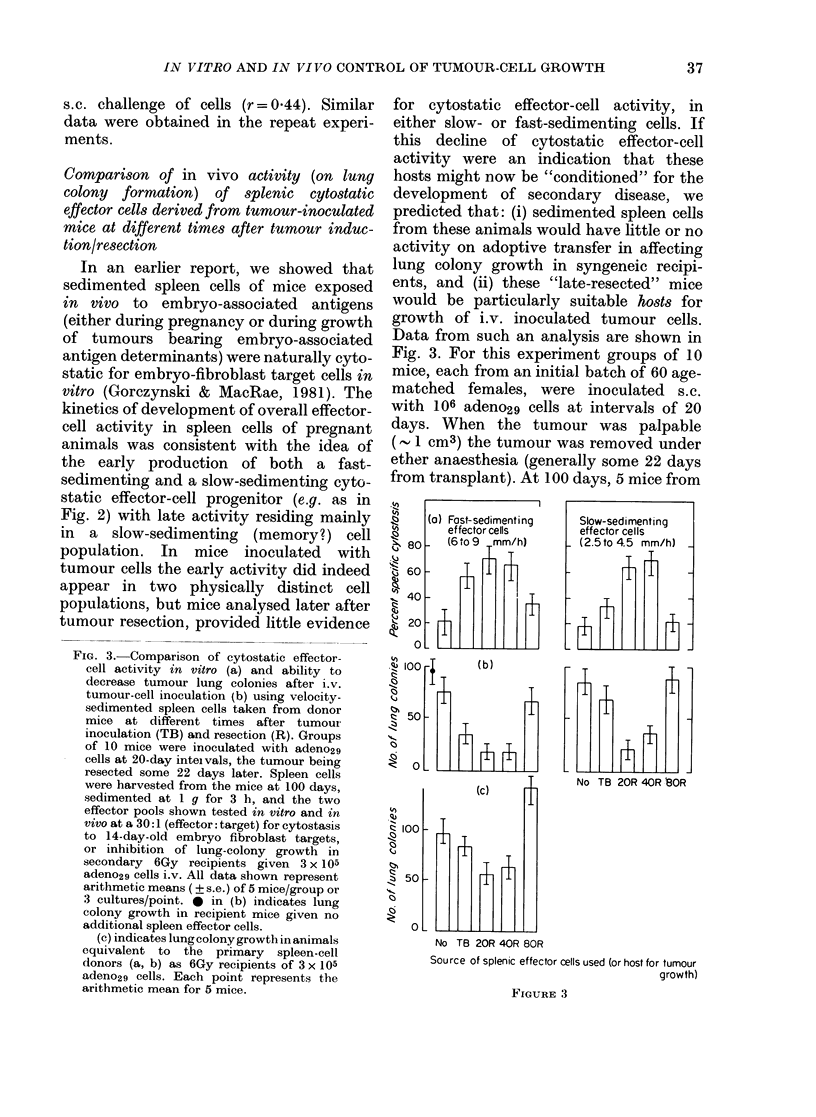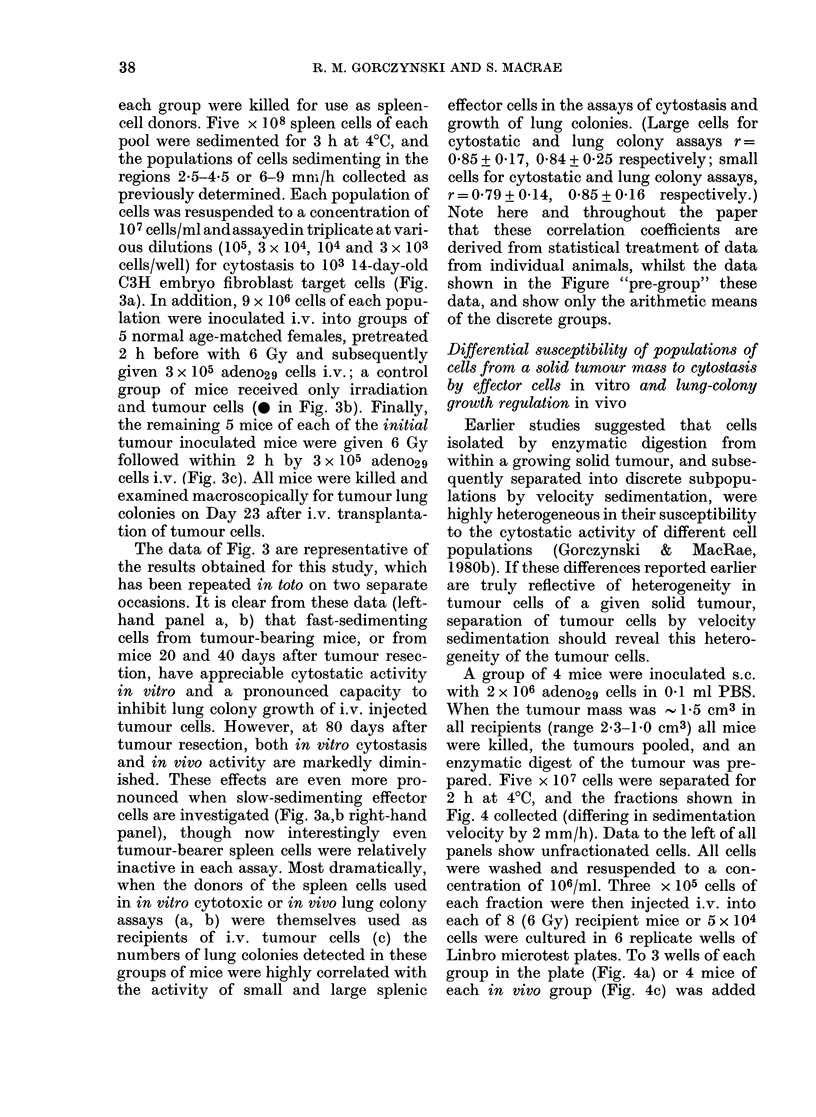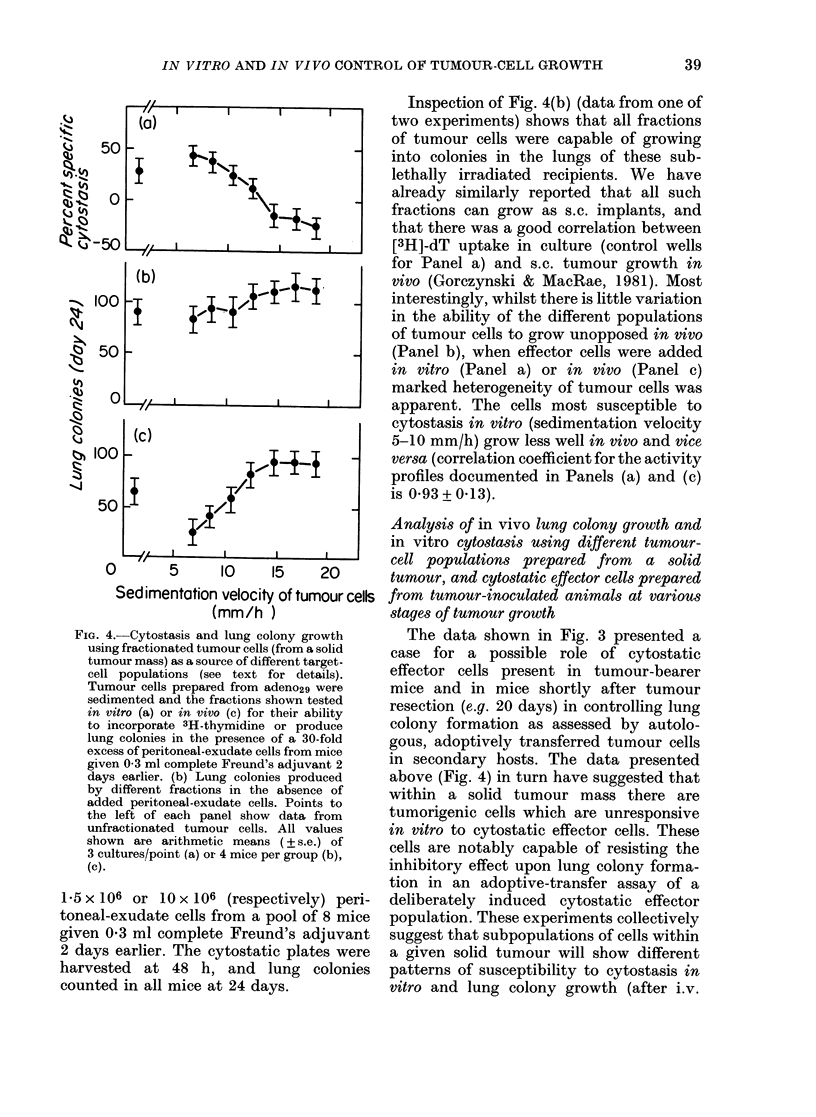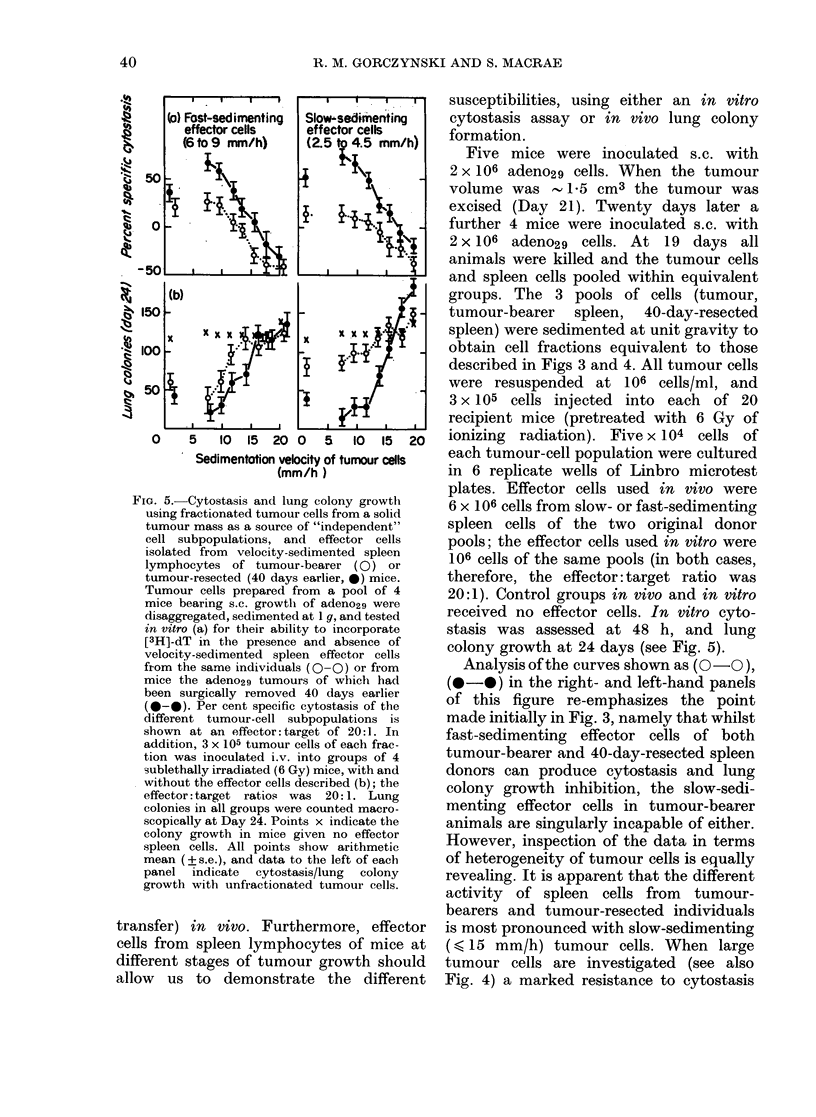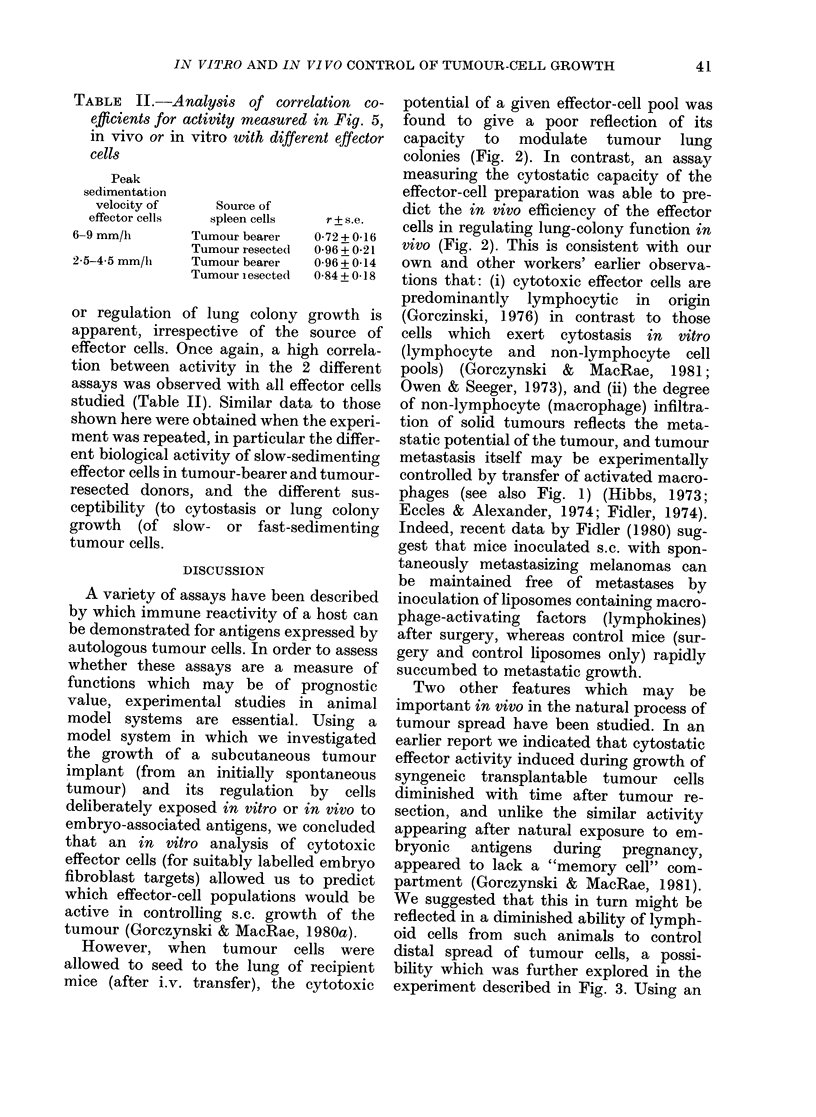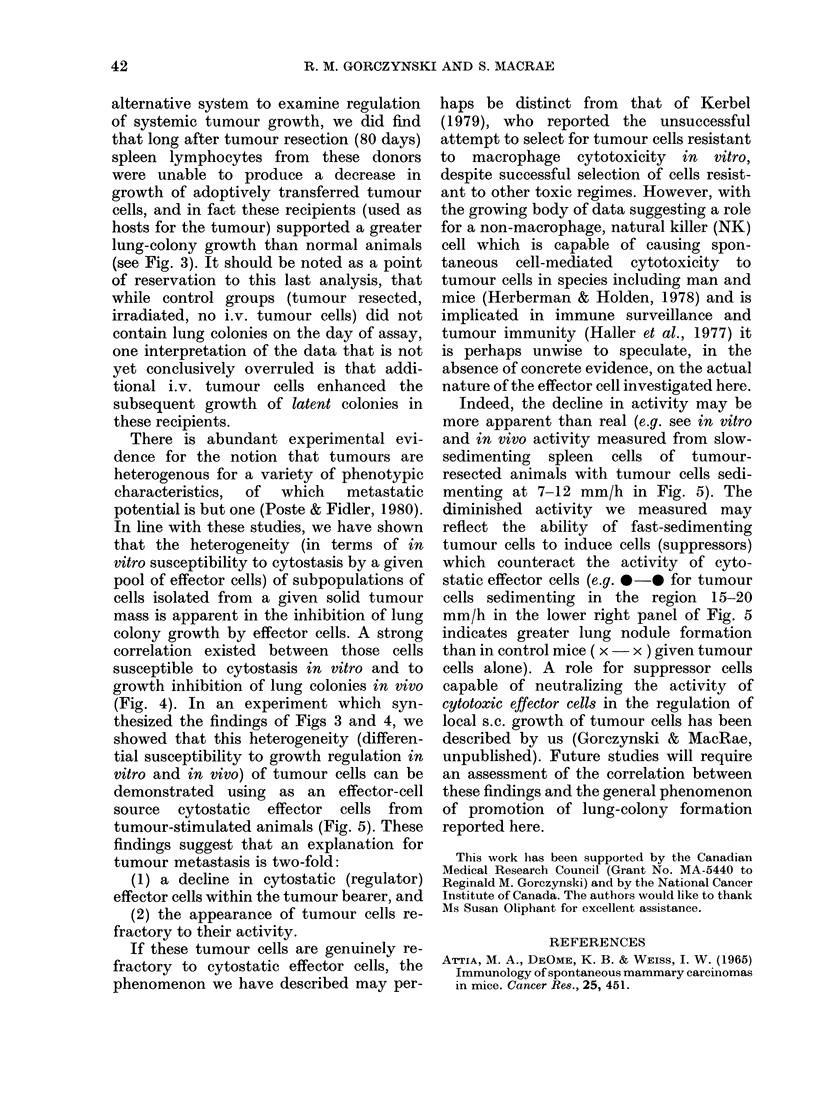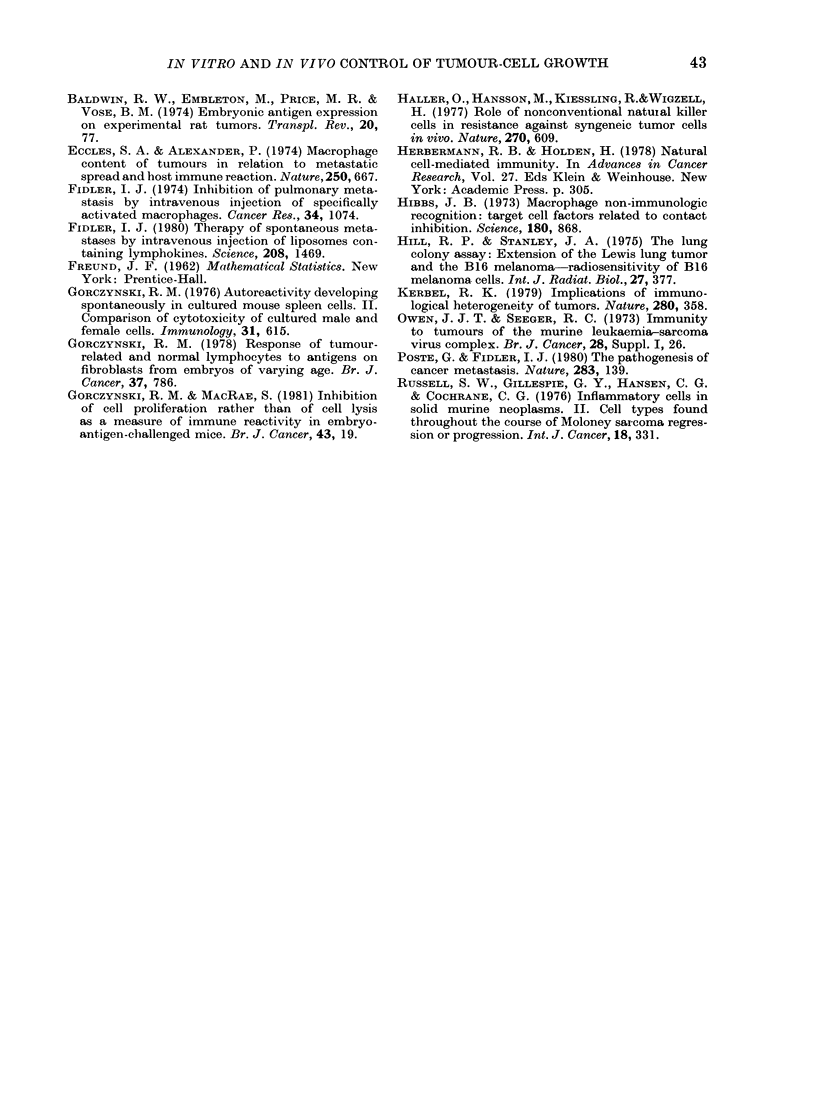# Tumour-cell susceptibility to cytotoxic or cytostatic effector cells in vitro and the regulation of tumour-cell growth in vivo.

**DOI:** 10.1038/bjc.1981.5

**Published:** 1981-01

**Authors:** R. M. Gorczynski, S. MacRae

## Abstract

Tumour-cell growth in lung nodules after i.v. transfer to sublethally irradiated mice has been followed after adoptive transfer of different populations of lymphoid cells. Spleen cells deliberately immunized in vitro and in vivo against stimulator cells bearing embryo-associated antigens and which are cytostatic in vitro for targets bearing such antigens, can diminish the number of lung nodules found after i.v. transfer. In contrast, cytotoxic (in vitro) spleen cells, while capable of diminishing local (s.c.) growth of tumour cells, cannot control systemic tumour growth. Within a given solid tumour mass, the subpopulations resistant to cytostatic effector cells in vitro are the ones most likely to produce lung colonies after adoptive transfer in vivo, though they show no more local (s.c.) growth than to cytostatic-sensitive cells in vivo.


*Br. J. Cancer* (1981) **43,** 32

### TUMOUR-CELL SUSCEPTIBILITY TO CYTOTOXIC OR CYTOSTATIC EFFECTOR CELLS *IN VITRO* AND THE REGULATION OF TUMOUR-CELL GROWTH *IN VIVO*

R. M. GORCZYNSKI* AND S. MACRAE

*From the Ontario Cancer Institute, Toronto M4X 1K9, Ontario, Canada*

Received 2 July 1980 Accepted 10 October 1980

**Summary.—Tumour-cell growth in lung nodules after i.v. transfer to sublethally irradiated mice has been followed after adoptive transfer of different populations of lymphoid cells. Spleen cells deliberately immunized *in vitro* and *in vivo* against stimulator cells bearing embryo-associated antigens and which are cytostatic *in vitro* for targets bearing such antigens, can diminish the number of lung nodules found after i.v. transfer. In contrast, cytotoxic (*in vitro*) spleen cells, while capable of diminishing local (s.c.) growth of tumour cells, cannot control systemic tumour growth. Within a given solid tumour mass, the subpopulations resistant to cytostatic effector cells *in vitro* are the ones most likely to produce lung colonies after adoptive transfer *in vivo*, though they show no more local (s.c.) growth than to cytostatic-sensitive cells *in vivo*.**

EARLIER WORK from this and other laboratories has established that embryo-immunized lymphocyte populations can confer protection (growth retardation) against s.c. implants of tumour cells in syngeneic hosts, and can decrease lung metastases in a rat hepatoma system (Gorczynski & MacRae, unpublished and 1981; Baldwin *et al.*, 1974). There is evidence in the literature that the state of macrophage activation, and the infiltration of such cells into the tumour mass, is correlated with metastases in the autologous host (Hibbs, 1973; Eccles & Alexander, 1974). In contrast, our studies have suggested that lymphoid cells, the activity of which can also be assessed in a 48h *in vitro* microcytotoxicity test, are the predominant effector-cell populations in regulating local (s.c.) growth of tumour cells (Gorczynski & MacRae, unpublished). An alternative cytotasis assay, also performed over a 48h culture period, detects, in addition to cytotoxic lymphocytes, other effector-cell populations (adherent, non-$\theta$-bearing) which also appear after deliberate exposure (during pregnancy or growth of tumours bearing neoantigens) of spleen cells to embryo-associated antigens *in vitro* or *in vivo* (Gorczynski & MacRae, 1981).

We have analysed the kinetics of appearance and loss of cytostatic effector-cell activity in the spleen of tumour-bearing and tumour-resected mice, and the heterogeneity of sensitivity of tumour-cell subpopulations (within one solid tumour mass) to cytostasis by a given activated cell population (Gorczynski & MacRae, 1981). These data suggested that our cytostasis assay may enable us to determine:

(i) the events in the lymphoid hierarchy of the host which may encourage the development of secondary tumour growth, and

(ii) the cells within a given tumour which have a potential for metastasis.

Both of these questions have been approached in the experiments described below, by correlation of a series of analyses on 4 independent assays per-

* To whom all correspondence should be addressed.

formed with either a standard tumour-cell population and a variety of effector cells, or the converse. Three of these assays, *in vitro* cytotoxicity and cytostasis assays, and *in vivo* growth modulation of s.c. implants of tumour cells, have been described in detail elsewhere (Gorczynski, 1978; Gorczynski & MacRae, unpub. and 1981). The 4th assay, lung colony growth after i.v. inoculation of tumour cells into irradiated mice, is documented below. The data support the notion that the events associated with the regulation of cellular cytostasis for embryo-antigen-bearing target cells *in vitro* are important predictors of tumour spread *in vivo*.

### MATERIALS AND METHODS

*Mice.*—C3H/HeJ mice were obtained from the Jackson Laboratories, Bar Harbor, Maine. All mice were kept 5 to a cage and allowed food and water *ad libitum*.

*Tumours.*—Retired breeder mice from Jackson Laboratories were inspected twice weekly for the appearance of spontaneous tumours. All data reported below were obtained with experiments using cells from one such transplantable tumour, $adeno_{29}$, a mammary adenocarcinoma (Department of Histology/Pathology, Princess Margaret Hospital). However, it is important to note that, without exception, similar results have been obtained using other such spontaneously appearing tumours ($adeno_{11}$, $adeno_{32}$, $adeno_{36}$).

Neoplasms were disaggregated with a mixture of 2–5% trypsin solution (Grand Island Biologicals, New York), crude collagenase (CLS II, 14 u/mg; Worthington Biochemicals, Freehold, New Jersey) and deoxyribonuclease (DNase I, B grade, $7 \times 10^4$ Dornase u/mg; Calbiochem, San Diego, California). All enzymes were used at a concentration of 0·1 $\mu$g/ml, and the tumour cells were harvested from the digestion flask for 90 min (at 30-min intervals). The yield from the initial primary $adeno_{29}$ tumour was $15 \times 10^7$ cells (Gorczynski, 1978; Russell *et al.*, 1976). $14 \times 10^7$ cells were frozen at $-70°C$ in $\alpha$-MEM containing dimethyl sulphoxide (DMSO) (15%) and foetal calf serum (30%) in aliquots of $3 \times 10^6$ cells (concentration $1{\cdot}5 \times 10^6$/ml). All experiments reported below used as starting material the cells prepared from an s.c. tumour produced in normal (8-week) female mice upon transplantation of $2 \times 10^6$ cells recovered from this frozen stock (mean recovery of viable cells on thawing was $40 \pm 10\%$)—the tumour grew to a mean volume of 1·8 cm$^3$ ($\pm 0{\cdot}3$ cm$^3$) after 20 days of transplantation of "thawed" primary cells.

Tumour resection was performed under ether anaesthesia as described in individual experiments. Tumour volume was measured with calipers, the volume being assessed by the formula: volume $= 0{\cdot}4ab^2$ where $a$ is the maximum dimension of the tumour and $b$ is the diameter at right angles to $a$ (Attia *et al.*, 1965).

*Preparation of embryo fibroblasts, spleen cells and techniques of velocity sedimentation and cell culture.*—These procedures were performed as described elsewhere (Gorczynski, 1978).

*Microcytotoxicity and cytostasis assays* (Gorczynski, 1978; Gorczynski & MacRae, 1981).—In brief, $2 \times 10^3$ embryo fibroblast target cells (either pre-labelled with $^3H$-proline for cytotoxicity assay, or post-labelled at 48 h with [$^3H$]-dT for cytostasis assay) were dispersed in 100 $\mu$l into wells of a 96-well Linbro microtitre plate. After allowing 3 h for the target cells to adhere, effector cells were added in a final volume of 200 $\mu$l at different concentrations to the well. All groups of varying effector:target ratios were set up in triplicate. Control groups contained only medium added to the targets (spontaneous cytotoxicity/cytostasis) or water (total releasable ct/min—cytotoxicity: maximum growth inhibition—cytostasis). At 48 h plates in the cytotoxicity assay were centrifuged at 500 $g$ for 5 min at 4°C; 100 $\mu$l of the supernatant in each well was dissolved in 5 ml Aquasol (New England Nuclear, Boston, Mass.) and the samples counted in a well-type scintillation counter. Percent specific cytotoxicity was measured as:

$$100 \times \frac{\text{ct/min experimental} - \text{ct/min spontaneous}}{\text{ct/min } H_2O - \text{ct/min spontaneous}}$$

At 48 h wells in the plates used for the cytostasis assay were washed $\times 3$ with sterile warm PBS, and 200 $\mu$l of [$^3H$]-dT in $\alpha$-MEM medium with 10% foetal calf serum ($\alpha F_{10}$) (0·5 $\mu$Ci/well) was added per well. The plates were returned to a humidified $CO_2$

incubator for 8 h when the wells were washed thoroughly and air-dried. The contents in each well were dissolved in 1·0M NaOH, transferred to scintillation vials, neutralized with 1·0M HCl, dissolved in 5 ml Aquasol and counted as above. Percent specific cytostasis was calculated as:

$$100 \times \frac{\text{ct/min spontaneous} - \text{ct/min experimental}}{\text{ct/min spontaneous} - \text{ct/min } H_2O}$$

As described elsewhere, this is an accurate reflection of cytostatic activity only in the absence of cytotoxicity from the population under test (Gorczynski & MacRae, 1981).

In vivo *assay for anti-tumour effector cells.*—An *in vivo* assay which detects cells able to regulate s.c. growth of implanted tumour cells is described elsewhere (Gorczynski & MacRae, unpublished). In brief, mice were lethally irradiated (9·5 Gy) and transplanted i.v. with $5 \times 10^6$ syngeneic marrow cells. Putative effector cells were also transferred i.v. at this time. Seven days after irradiation all mice were inoculated s.c. with $10^6$ tumour cells in 0·15 ml PBS. Tumour volume was measured 3 times a week after the tumour became palpable.

Recipient mice were irradiated (6 Gy) 6 h before i.v. tumour-cell inoculation, and received putative effector cells 2 h after irradiation. The animals were killed 21–24 days after irradiation, which generally marked the first evidence for respiratory distress. The lungs were removed and placed into Bouin's solution, and lung colonies counted macroscopically. At least 4 mice were used per group.

*Statistical analysis.*—Statistical comparison of experimentally determined parameters for different groups of animals (or cell populations derived from them) were performed using a non-parametric Mann–Whitney test (Freund, 1962).

### RESULTS

### *Quantitation of inhibition of growth of tumour lung nodules as a function of effector cells transferred*

In a series of earlier studies we have developed quantitative long-term (48h) assays *in vitro* by which cytotoxicity or cytostasis of an effector population for target cells bearing embryo-associated antigenic determinants can be measured (Gorczynski, 1978; Gorczynski & MacRae, 1981). Furthermore, we have shown that similar quantitative analysis can be used to assess the regulation of growth of s.c. tumour-cell implants by different effector-cell populations (Gorczynski & MacRae, unpublished). Effector cells in cytostasis

FIG. 1.—Tumour-cell dose–response curve for lung colonies after i.v. inoculation of tumour cells (a) and effect of activated peritoneal–exudate cells on lung-colony formation (b). All mice given tumour cells ($adeno_{29}$) i.v. were pretreated with 6 Gy whole-body irradiation 2 h before inoculation.

(a) Tumour cells were given alone (in 0·5 ml PBS, ×) or with $2 \times 10^7$ irradiated (20 Gy) normal spleen cells as an inert carrier population (●). Five mice were used per group and lung colonies were counted macroscopically 25 days later.

(b) All mice received $3 \times 10^5$ tumour cells (mixed with $2 \times 10^7$ irradiated carrier spleen cells) along with varying numbers of peritoneal-exudate cells harvested from mice given 0·3 ml complete Freund's adjuvant 2 days earlier (×). The peritoneal–exudate cells were also tested in triplicate at different dilutions for their ability to cause cytostasis of $2 \times 10^3$ 14-day C3H embryo fibroblast target cells in a standard *in vitro* assay (●). All points represent arithmetic means ± s.e.

and cytotoxicity assays were not identical cell populations. In the absence of a highly metastatic spontaneous adenocarcinoma, we have resorted to another means of studying the regulation of growth of tumour cells which escape into the systemic circulation, by deliberately transferring different i.v. doses of tumour cells ($3 \times 10^4$ or $3 \times 10^5$) in 0·5 ml PBS to groups of 5 sublethally irradiated (6 Gy) recipients—Fig. 1(a). An inert "carrier" cell population (20 Gy syngeneic normal spleen cells, $2 \times 10^7$ per recipient) was included in some recipients (●—●, compare with ×—×, no carrier cells) to study whether "seeding" of tumour cells to the lung was more effective at the various tumour-cell dilutions in the presence of such a carrier (Hill & Stanley, 1975). The data of this panel (a) of Fig. 1 indicate that, in the presence of an inert carrier-cell population, the number of tumour lung nodules counted macroscopically at Day 25 after transfer of tumour cells was a function of the number of tumour cells inoculated. Similar results were obtained in 3 repeat experiments (data not shown).

When peritoneal-exudate cells were taken from mice 48 h after i.p. inoculation of 0·3 ml complete Freund's adjuvant, and examined for their ability to affect tumour lung colony growth or cause cytostasis of embryo fibroblast targets *in vitro*, the data of Fig. 1(b) were obtained. As can be seen from this Figure, cells with cytostatic activity (●—●) exert appreciable inhibition of lung colony formation when given i.v. along with tumour cells. In two repeat experiments in which we inoculated this large tumour-cell dose ($3\text{–}5 \times 10^5$) *without carrier cells*, we again found a quantitative inhibition of colony formation by activated peritoneal-exudate cells (data not shown). Thus, unless the experimental protocol involved variations in the

FIGURE 2.

FIG. 2.—Comparison of *in vitro* and *in vivo* assays of anti-tumour and anti-embryo effector-cell activity using spleen lymphocytes obtained from 5-day cultures of normal C3H/HeJ female cells. Spleen cells harvested from cultures initially containing $5 \times 10^8$ cells were sedimented for 4 h at 4°C. The fractions shown were harvested and assayed (along with an unfractionated control sample at extreme left) for activity at various dilutions in cytotoxicity (a) and cytostasis (b) tests with 14-day-old C3H embryo fibroblast targets or *in vivo* for inhibition of growth of s.c. (c) and i.v. (d) transplanted cells from a spontaneously appearing tumour, $adeno_{29}$. Data from the *in vitro* tests are for an equivalent effector: target ratio for unfractionated cells of 23:1; for *in vivo* assays the data points represent an equivalent effector:target ratio for unfractionated cells of 25:1 (s.c.) or 8:1 (i.v.). All values shown represent the arithmetic mean of 3 cultures per point, or 5 mice per group (*in vivo* assay).

dose of tumour cells in the inocula, irradiated spleen carrier cells were omitted from the experiments described below.

*Correlation between* in vitro *and* in vivo *assays for detecting effector cells regulating the growth of cells with embryo-associated antigenic determinants*

We have described both *in vitro* (cytotoxicity and cytostasis) and *in vivo* (regulation of growth of s.c. tumour implants; inhibition of lung colony growth after i.v. inoculation) assays which detect effector cells expressing activity against spontaneously appearing adenocarcinoma target cells. Spleen lymphoid cells precultured for 5 days are known to express activity in the first 3 assays mentioned, though the *in vitro* cytotoxicity assay was found to be better correlated with regulation of growth of s.c. tumour implants than the cytostasis assay (Gorczynski & MacRae, unpublished). In order to assess the ability of cultured spleen cells to affect tumour-cell growth as lung colonies, we cultured $5 \times 10^8$ female spleen lymphoid cells in Falcon culture flasks (75 $cm^2$ growth area; $10^8$ cells per flask in 100 ml $\alpha F_{10}$). After 5 days the cells recovered from the flasks ($2 \times 10^8$) were pooled, washed (1000 rev/min for 5 min at 4°C) and resuspended in 35 ml 0·3% BSA in PBS. Thirty ml of the cells were sedimented for 4 h at 4°C over a gradient ranging from 0·6% BSA to 2% BSA in PBS, and cells differing in sedimentation velocity by 1 mm/h were collected. The various fractions and an unfractionated control sample were tested at various dilutions (0·05%, 0·15% and 0·5% of the cells per fraction) in triplicate *in vitro*, using $2 \times 10^3$ 14-day-old C3H embryo fibroblast cells/well in Linbro microtest wells. Target cells were prelabelled with $^3$H-proline or post-labelled with [$^3$H]-dT according to whether cyto toxicity or cytostasis assays were to be performed. Data shown in Fig. 2 represent cytotoxicity and cytostasis for 0·15% of the cells/fraction (representing an unfractionated effector:target of 23:1).

In addition to these *in vitro* assays, 8% of the cells of each fraction (an unfractionated equivalent of $2 \cdot 5 \times 10^6$ cells) was injected i.v. into 2 groups of 5 per group C3H/HeJ female mice, either given 6 Gy or 9·5 Gy and $5 \times 10^6$ syngeneic marrow cells 2 h earlier. The group of 5 mice receiving 6 Gy were given $3 \times 10^5$ tumour cells i.v. 6 h after spleen-cell transfer, the other group receiving $10^6$ tumour cells s.c. 7 days later. All tumour cells used were from a spontaneously appearing adenocarcinoma (adeno$_{29}$). Lung colonies were examined in the former groups at 21 days, and tumour growth in the latter groups measured with calipers every 3 days after the appearance of palpable tumour. The data for one experiment (of 3) of this type are shown in Fig. 2 (tumour volumes for these groups were assessed at Day 38).

Visual inspection of these data, coupled with statistical analysis of the coefficient of correlation for the activity profiles represented by individual panels (see Table I) provides evidence that whilst activity in an *in vitro* cytotoxicity test is better correlated with inhibition of local s.c. growth of tumours than appearance of lung nodules after i.v. injection of tumour cells ($r = 0 \cdot 90$ *vs* $r = 0 \cdot 57$) assays for cytostatic activity appear to detect preferentially those cells capable of regulating an i.v. challenge of tumour cells ($r = 0 \cdot 99$) rather than those regulating an

TABLE I.—*Analysis of correlation coefficients* (±*s.e.*) *of sedimentation profiles shown in Fig.* 2

| Assay performed→ ↓ | % cytostasis | Tumour vol. (s.c. growth) | No. of lung colonies |
|---|---|---|---|
| % cytotoxicity | −0·63±0·15 | −0·90±0·16 | 0·57±0·14 |
| % cytostasis | | 0·44±0·14 | −0·99±0·21 |
| Tumour volume (s.c. growth) | | | 0·39±0·12 |

s.c. challenge of cells ($r = 0{\cdot}44$). Similar data were obtained in the repeat experiments.

*Comparison of* in vivo *activity (on lung colony formation) of splenic cytostatic effector cells derived from tumour-inoculated mice at different times after tumour induction/resection*

In an earlier report, we showed that sedimented spleen cells of mice exposed *in vivo* to embryo-associated antigens (either during pregnancy or during growth of tumours bearing embryo-associated antigen determinants) were naturally cytostatic for embryo-fibroblast target cells *in vitro* (Gorczynski & MacRae, 1981). The kinetics of development of overall effector-cell activity in spleen cells of pregnant animals was consistent with the idea of the early production of both a fast-sedimenting and a slow-sedimenting cytostatic effector-cell progenitor (*e.g.* as in Fig. 2) with late activity residing mainly in a slow-sedimenting (memory?) cell population. In mice inoculated with tumour cells the early activity did indeed appear in two physically distinct cell populations, but mice analysed later after tumour resection, provided little evidence for cytostatic effector-cell activity, in either slow- or fast-sedimenting cells. If this decline of cytostatic effector-cell activity were an indication that these hosts might now be "conditioned" for the development of secondary disease, we predicted that: (i) sedimented spleen cells from these animals would have little or no activity on adoptive transfer in affecting lung colony growth in syngeneic recipients, and (ii) these "late-resected" mice would be particularly suitable ***hosts*** for growth of i.v. inoculated tumour cells. Data from such an analysis are shown in Fig. 3. For this experiment groups of 10 mice, each from an initial batch of 60 age-matched females, were inoculated s.c. with $10^6$ adeno$_{29}$ cells at intervals of 20 days. When the tumour was palpable ($\sim 1$ cm$^3$) the tumour was removed under ether anaesthesia (generally some 22 days from transplant). At 100 days, 5 mice from

FIGURE 3

FIG. 3.—Comparison of cytostatic effector-cell activity *in vitro* (a) and ability to decrease tumour lung colonies after i.v. tumour-cell inoculation (b) using velocity-sedimented spleen cells taken from donor mice at different times after tumour inoculation (TB) and resection (R). Groups of 10 mice were inoculated with adeno$_{29}$ cells at 20-day intervals, the tumour being resected some 22 days later. Spleen cells were harvested from the mice at 100 days, sedimented at 1 *g* for 3 h, and the two effector pools shown tested *in vitro* and *in vivo* at a 30:1 (effector:target) for cytostasis to 14-day-old embryo fibroblast targets, or inhibition of lung-colony growth in secondary 6Gy recipients given $3 \times 10^5$ adeno$_{29}$ cells i.v. All data shown represent arithmetic means ($\pm$s.e.) of 5 mice/group or 3 cultures/point. ● in (b) indicates lung colony growth in recipient mice given no additional spleen effector cells.

(c) indicates lung colony growth in animals equivalent to the primary spleen-cell donors (a, b) as 6Gy recipients of $3 \times 10^5$ adeno$_{29}$ cells. Each point represents the arithmetic mean for 5 mice.

each group were killed for use as spleen-cell donors. Five $\times 10^8$ spleen cells of each pool were sedimented for 3 h at 4°C, and the populations of cells sedimenting in the regions 2·5–4·5 or 6–9 mm/h collected as previously determined. Each population of cells was resuspended to a concentration of $10^7$ cells/ml and assayed in triplicate at various dilutions ($10^5$, $3 \times 10^4$, $10^4$ and $3 \times 10^3$ cells/well) for cytostasis to $10^3$ 14-day-old C3H embryo fibroblast target cells (Fig. 3a). In addition, $9 \times 10^6$ cells of each population were inoculated i.v. into groups of 5 normal age-matched females, pretreated 2 h before with 6 Gy and subsequently given $3 \times 10^5$ $adeno_{29}$ cells i.v.; a control group of mice received only irradiation and tumour cells (● in Fig. 3b). Finally, the remaining 5 mice of each of the *initial* tumour inoculated mice were given 6 Gy followed within 2 h by $3 \times 10^5$ $adeno_{29}$ cells i.v. (Fig. 3c). All mice were killed and examined macroscopically for tumour lung colonies on Day 23 after i.v. transplantation of tumour cells.

The data of Fig. 3 are representative of the results obtained for this study, which has been repeated *in toto* on two separate occasions. It is clear from these data (left-hand panel a, b) that fast-sedimenting cells from tumour-bearing mice, or from mice 20 and 40 days after tumour resection, have appreciable cytostatic activity *in vitro* and a pronounced capacity to inhibit lung colony growth of i.v. injected tumour cells. However, at 80 days after tumour resection, both *in vitro* cytostasis and *in vivo* activity are markedly diminished. These effects are even more pronounced when slow-sedimenting effector cells are investigated (Fig. 3a,b right-hand panel), though now interestingly even tumour-bearer spleen cells were relatively inactive in each assay. Most dramatically, when the donors of the spleen cells used in *in vitro* cytotoxic or *in vivo* lung colony assays (a, b) were themselves used as recipients of i.v. tumour cells (c) the numbers of lung colonies detected in these groups of mice were highly correlated with the activity of small and large splenic effector cells in the assays of cytostasis and growth of lung colonies. (Large cells for cytostatic and lung colony assays $r = 0{\cdot}85 \pm 0{\cdot}17$, $0{\cdot}84 \pm 0{\cdot}25$ respectively; small cells for cytostatic and lung colony assays, $r = 0{\cdot}79 \pm 0{\cdot}14$, $0{\cdot}85 \pm 0{\cdot}16$ respectively.) Note here and throughout the paper that these correlation coefficients are derived from statistical treatment of data from individual animals, whilst the data shown in the Figure "pre-group" these data, and show only the arithmetic means of the discrete groups.

*Differential susceptibility of populations of cells from a solid tumour mass to cytostasis by effector cells* in vitro *and lung-colony growth regulation* in vivo

Earlier studies suggested that cells isolated by enzymatic digestion from within a growing solid tumour, and subsequently separated into discrete subpopulations by velocity sedimentation, were highly heterogeneous in their susceptibility to the cytostatic activity of different cell populations (Gorczynski & MacRae, 1980b). If these differences reported earlier are truly reflective of heterogeneity in tumour cells of a given solid tumour, separation of tumour cells by velocity sedimentation should reveal this heterogeneity of the tumour cells.

A group of 4 mice were inoculated s.c. with $2 \times 10^6$ $adeno_{29}$ cells in 0·1 ml PBS. When the tumour mass was $\sim 1{\cdot}5$ cm$^3$ in all recipients (range 2·3–1·0 cm$^3$) all mice were killed, the tumours pooled, and an enzymatic digest of the tumour was prepared. Five $\times 10^7$ cells were separated for 2 h at 4°C, and the fractions shown in Fig. 4 collected (differing in sedimentation velocity by 2 mm/h). Data to the left of all panels show unfractionated cells. All cells were washed and resuspended to a concentration of $10^6$/ml. Three $\times 10^5$ cells of each fraction were then injected i.v. into each of 8 (6 Gy) recipient mice or $5 \times 10^4$ cells were cultured in 6 replicate wells of Linbro microtest plates. To 3 wells of each group in the plate (Fig. 4a) or 4 mice of each *in vivo* group (Fig. 4c) was added

FIG. 4.—Cytostasis and lung colony growth using fractionated tumour cells (from a solid tumour mass) as a source of different target-cell populations (see text for details). Tumour cells prepared from adeno$_{29}$ were sedimented and the fractions shown tested *in vitro* (a) or *in vivo* (c) for their ability to incorporate $^3$H-thymidine or produce lung colonies in the presence of a 30-fold excess of peritoneal-exudate cells from mice given 0·3 ml complete Freund's adjuvant 2 days earlier. (b) Lung colonies produced by different fractions in the absence of added peritoneal-exudate cells. Points to the left of each panel show data from unfractionated tumour cells. All values shown are arithmetic means ($\pm$s.e.) of 3 cultures/point (a) or 4 mice per group (b), (c).

$1{\cdot}5\times10^6$ or $10\times10^6$ (respectively) peritoneal-exudate cells from a pool of 8 mice given 0·3 ml complete Freund's adjuvant 2 days earlier. The cytostatic plates were harvested at 48 h, and lung colonies counted in all mice at 24 days.

Inspection of Fig. 4(b) (data from one of two experiments) shows that all fractions of tumour cells were capable of growing into colonies in the lungs of these sub-lethally irradiated recipients. We have already similarly reported that all such fractions can grow as s.c. implants, and that there was a good correlation between [$^3$H]-dT uptake in culture (control wells for Panel a) and s.c. tumour growth *in vivo* (Gorczynski & MacRae, 1981). Most interestingly, whilst there is little variation in the ability of the different populations of tumour cells to grow unopposed *in vivo* (Panel b), when effector cells were added *in vitro* (Panel a) or *in vivo* (Panel c) marked heterogeneity of tumour cells was apparent. The cells most susceptible to cytostasis *in vitro* (sedimentation velocity 5–10 mm/h) grow less well *in vivo* and *vice versa* (correlation coefficient for the activity profiles documented in Panels (a) and (c) is $0{\cdot}93\pm0{\cdot}13$).

*Analysis of* in vivo *lung colony growth and* in vitro *cytostasis using different tumour-cell populations prepared from a solid tumour, and cytostatic effector cells prepared from tumour-inoculated animals at various stages of tumour growth*

The data shown in Fig. 3 presented a case for a possible role of cytostatic effector cells present in tumour-bearer mice and in mice shortly after tumour resection (*e.g.* 20 days) in controlling lung colony formation as assessed by autologous, adoptively transferred tumour cells in secondary hosts. The data presented above (Fig. 4) in turn have suggested that within a solid tumour mass there are tumorigenic cells which are unresponsive *in vitro* to cytostatic effector cells. These cells are notably capable of resisting the inhibitory effect upon lung colony formation in an adoptive-transfer assay of a deliberately induced cytostatic effector population. These experiments collectively suggest that subpopulations of cells within a given solid tumour will show different patterns of susceptibility to cytostasis *in vitro* and lung colony growth (after i.v.

FIG. 5.—Cytostasis and lung colony growth using fractionated tumour cells from a solid tumour mass as a source of "independent" cell subpopulations, and effector cells isolated from velocity-sedimented spleen lymphocytes of tumour-bearer (○) or tumour-resected (40 days earlier, ●) mice. Tumour cells prepared from a pool of 4 mice bearing s.c. growth of $adeno_{29}$ were disaggregated, sedimented at 1 *g*, and tested *in vitro* (a) for their ability to incorporate [$^3$H]-dT in the presence and absence of velocity-sedimented spleen effector cells from the same individuals (○–○) or from mice the $adeno_{29}$ tumours of which had been surgically removed 40 days earlier (●–●). Per cent specific cytostasis of the different tumour-cell subpopulations is shown at an effector: target of 20:1. In addition, $3 \times 10^5$ tumour cells of each fraction was inoculated i.v. into groups of 4 sublethally irradiated (6 Gy) mice, with and without the effector cells described (b); the effector: target ratios was 20:1. Lung colonies in all groups were counted macroscopically at Day 24. Points × indicate the colony growth in mice given no effector spleen cells. All points show arithmetic mean (±s.e.), and data to the left of each panel indicate cytostasis/lung colony growth with unfractionated tumour cells.

transfer) *in vivo*. Furthermore, effector cells from spleen lymphocytes of mice at different stages of tumour growth should allow us to demonstrate the different susceptibilities, using either an *in vitro* cytostasis assay or *in vivo* lung colony formation.

Five mice were inoculated s.c. with $2 \times 10^6$ $adeno_{29}$ cells. When the tumour volume was $\sim 1{\cdot}5$ cm$^3$ the tumour was excised (Day 21). Twenty days later a further 4 mice were inoculated s.c. with $2 \times 10^6$ $adeno_{29}$ cells. At 19 days all animals were killed and the tumour cells and spleen cells pooled within equivalent groups. The 3 pools of cells (tumour, tumour-bearer spleen, 40-day-resected spleen) were sedimented at unit gravity to obtain cell fractions equivalent to those described in Figs 3 and 4. All tumour cells were resuspended at $10^6$ cells/ml, and $3 \times 10^5$ cells injected into each of 20 recipient mice (pretreated with 6 Gy of ionizing radiation). Five $\times 10^4$ cells of each tumour-cell population were cultured in 6 replicate wells of Linbro microtest plates. Effector cells used *in vivo* were $6 \times 10^6$ cells from slow- or fast-sedimenting spleen cells of the two original donor pools; the effector cells used *in vitro* were $10^6$ cells of the same pools (in both cases, therefore, the effector: target ratio was 20:1). Control groups *in vivo* and *in vitro* received no effector cells. *In vitro* cytostasis was assessed at 48 h, and lung colony growth at 24 days (see Fig. 5).

Analysis of the curves shown as (○—○), (●—●) in the right- and left-hand panels of this figure re-emphasizes the point made initially in Fig. 3, namely that whilst fast-sedimenting effector cells of both tumour-bearer and 40-day-resected spleen donors can produce cytostasis and lung colony growth inhibition, the slow-sedimenting effector cells in tumour-bearer animals are singularly incapable of either. However, inspection of the data in terms of heterogeneity of tumour cells is equally revealing. It is apparent that the different activity of spleen cells from tumour-bearers and tumour-resected individuals is most pronounced with slow-sedimenting ($\leqslant 15$ mm/h) tumour cells. When large tumour cells are investigated (see also Fig. 4) a marked resistance to cytostasis

TABLE II.—*Analysis of correlation coefficients for activity measured in Fig.* 5, in vivo *or* in vitro *with different effector cells*

| Peak sedimentation velocity of effector cells | Source of spleen cells | $r \pm$ s.e. |
|---|---|---|
| 6–9 mm/h | Tumour bearer | 0·72 ± 0·16 |
| | Tumour resected | 0·96 ± 0·21 |
| 2·5–4·5 mm/h | Tumour bearer | 0·96 ± 0·14 |
| | Tumour resected | 0·84 ± 0·18 |

or regulation of lung colony growth is apparent, irrespective of the source of effector cells. Once again, a high correlation between activity in the 2 different assays was observed with all effector cells studied (Table II). Similar data to those shown here were obtained when the experiment was repeated, in particular the different biological activity of slow-sedimenting effector cells in tumour-bearer and tumour-resected donors, and the different susceptibility (to cytostasis or lung colony growth (of slow- or fast-sedimenting tumour cells.

### DISCUSSION

A variety of assays have been described by which immune reactivity of a host can be demonstrated for antigens expressed by autologous tumour cells. In order to assess whether these assays are a measure of functions which may be of prognostic value, experimental studies in animal model systems are essential. Using a model system in which we investigated the growth of a subcutaneous tumour implant (from an initially spontaneous tumour) and its regulation by cells deliberately exposed *in vitro* or *in vivo* to embryo-associated antigens, we concluded that an *in vitro* analysis of cytotoxic effector cells (for suitably labelled embryo fibroblast targets) allowed us to predict which effector-cell populations would be active in controlling s.c. growth of the tumour (Gorczynski & MacRae, 1980*a*).

However, when tumour cells were allowed to seed to the lung of recipient mice (after i.v. transfer), the cytotoxic potential of a given effector-cell pool was found to give a poor reflection of its capacity to modulate tumour lung colonies (Fig. 2). In contrast, an assay measuring the cytostatic capacity of the effector-cell preparation was able to predict the *in vivo* efficiency of the effector cells in regulating lung-colony function *in vivo* (Fig. 2). This is consistent with our own and other workers' earlier observations that: (i) cytotoxic effector cells are predominantly lymphocytic in origin (Gorczinski, 1976) in contrast to those cells which exert cytostasis *in vitro* (lymphocyte and non-lymphocyte cell pools) (Gorczynski & MacRae, 1981; Owen & Seeger, 1973), and (ii) the degree of non-lymphocyte (macrophage) infiltration of solid tumours reflects the metastatic potential of the tumour, and tumour metastasis itself may be experimentally controlled by transfer of activated macrophages (see also Fig. 1) (Hibbs, 1973; Eccles & Alexander, 1974; Fidler, 1974). Indeed, recent data by Fidler (1980) suggest that mice inoculated s.c. with spontaneously metastasizing melanomas can be maintained free of metastases by inoculation of liposomes containing macrophage-activating factors (lymphokines) after surgery, whereas control mice (surgery and control liposomes only) rapidly succumbed to metastatic growth.

Two other features which may be important *in vivo* in the natural process of tumour spread have been studied. In an earlier report we indicated that cytostatic effector activity induced during growth of syngeneic transplantable tumour cells diminished with time after tumour resection, and unlike the similar activity appearing after natural exposure to embryonic antigens during pregnancy, appeared to lack a "memory cell" compartment (Gorczynski & MacRae, 1981). We suggested that this in turn might be reflected in a diminished ability of lymphoid cells from such animals to control distal spread of tumour cells, a possibility which was further explored in the experiment described in Fig. 3. Using an alternative system to examine regulation of systemic tumour growth, we did find that long after tumour resection (80 days) spleen lymphocytes from these donors were unable to produce a decrease in growth of adoptively transferred tumour cells, and in fact these recipients (used as hosts for the tumour) supported a greater lung-colony growth than normal animals (see Fig. 3). It should be noted as a point of reservation to this last analysis, that while control groups (tumour resected, irradiated, no i.v. tumour cells) did not contain lung colonies on the day of assay, one interpretation of the data that is not yet conclusively overruled is that additional i.v. tumour cells enhanced the subsequent growth of *latent* colonies in these recipients.

There is abundant experimental evidence for the notion that tumours are heterogenous for a variety of phenotypic characteristics, of which metastatic potential is but one (Poste & Fidler, 1980). In line with these studies, we have shown that the heterogeneity (in terms of *in vitro* susceptibility to cytostasis by a given pool of effector cells) of subpopulations of cells isolated from a given solid tumour mass is apparent in the inhibition of lung colony growth by effector cells. A strong correlation existed between those cells susceptible to cytostasis *in vitro* and to growth inhibition of lung colonies *in vivo* (Fig. 4). In an experiment which synthesized the findings of Figs 3 and 4, we showed that this heterogeneity (differential susceptibility to growth regulation *in vitro* and *in vivo*) of tumour cells can be demonstrated using as an effector-cell source cytostatic effector cells from tumour-stimulated animals (Fig. 5). These findings suggest that an explanation for tumour metastasis is two-fold:

(1) a decline in cytostatic (regulator) effector cells within the tumour bearer, and

(2) the appearance of tumour cells refractory to their activity.

If these tumour cells are genuinely refractory to cytostatic effector cells, the phenomenon we have described may perhaps be distinct from that of Kerbel (1979), who reported the unsuccessful attempt to select for tumour cells resistant to macrophage cytotoxicity *in vitro*, despite successful selection of cells resistant to other toxic regimes. However, with the growing body of data suggesting a role for a non-macrophage, natural killer (NK) cell which is capable of causing spontaneous cell-mediated cytotoxicity to tumour cells in species including man and mice (Herberman & Holden, 1978) and is implicated in immune surveillance and tumour immunity (Haller *et al.*, 1977) it is perhaps unwise to speculate, in the absence of concrete evidence, on the actual nature of the effector cell investigated here.

Indeed, the decline in activity may be more apparent than real (*e.g.* see *in vitro* and *in vivo* activity measured from slow-sedimenting spleen cells of tumour-resected animals with tumour cells sedimenting at 7–12 mm/h in Fig. 5). The diminished activity we measured may reflect the ability of fast-sedimenting tumour cells to induce cells (suppressors) which counteract the activity of cytostatic effector cells (*e.g.* ●—● for tumour cells sedimenting in the region 15–20 mm/h in the lower right panel of Fig. 5 indicates greater lung nodule formation than in control mice ( × — × ) given tumour cells alone). A role for suppressor cells capable of neutralizing the activity of *cytotoxic effector cells* in the regulation of local s.c. growth of tumour cells has been described by us (Gorczynski & MacRae, unpublished). Future studies will require an assessment of the correlation between these findings and the general phenomenon of promotion of lung-colony formation reported here.

This work has been supported by the Canadian Medical Research Council (Grant No. MA-5440 to Reginald M. Gorczynski) and by the National Cancer Institute of Canada. The authors would like to thank Ms Susan Oliphant for excellent assistance.

### REFERENCES

ATTIA, M. A., DEOME, K. B. & WEISS, I. W. (1965) Immunology of spontaneous mammary carcinomas in mice. *Cancer Res.*, **25**, 451.

BALDWIN, R. W., EMBLETON, M., PRICE, M. R. & VOSE, B. M. (1974) Embryonic antigen expression on experimental rat tumors. *Transpl. Rev.*, **20**, 77.

ECCLES, S. A. & ALEXANDER, P. (1974) Macrophage content of tumours in relation to metastatic spread and host immune reaction. *Nature*, **250**, 667.

FIDLER, I. J. (1974) Inhibition of pulmonary metastasis by intravenous injection of specifically activated macrophages. *Cancer Res.*, **34**, 1074.

FIDLER, I. J. (1980) Therapy of spontaneous metastases by intravenous injection of liposomes containing lymphokines. *Science*, **208**, 1469.

FREUND, J. F. (1962) *Mathematical Statistics*. New York: Prentice-Hall.

GORCZYNSKI, R. M. (1976) Autoreactivity developing spontaneously in cultured mouse spleen cells. II. Comparison of cytotoxicity of cultured male and female cells. *Immunology*, **31**, 615.

GORCZYNSKI, R. M. (1978) Response of tumour-related and normal lymphocytes to antigens on fibroblasts from embryos of varying age. *Br. J. Cancer*, **37**, 786.

GORCZYNSKI, R. M. & MACRAE, S. (1981) Inhibition of cell proliferation rather than of cell lysis as a measure of immune reactivity in embryo-antigen-challenged mice. *Br. J. Cancer*, **43**, 19.

HALLER, O., HANSSON, M., KIESSLING, R. & WIGZELL, H. (1977) Role of nonconventional natuıal killer cells in resistance against syngeneic tumor cells *in vivo*. *Nature*, **270**, 609.

HERBERMANN, R. B. & HOLDEN, H. (1978) Natural cell-mediated immunity. In *Advances in Cancer Research*, Vol. 27. Eds Klein & Weinhouse. New York: Academic Press. p. 305.

HIBBS, J. B. (1973) Macrophage non-immunologic recognition: target cell factors related to contact inhibition. *Science*, **180**, 868.

HILL, R. P. & STANLEY, J. A. (1975) The lung colony assay: Extension of the Lewis lung tumor and the B16 melanoma—radiosensitivity of B16 melanoma cells. *Int. J. Radiat. Biol.*, **27**, 377.

KERBEL, R. K. (1979) Implications of immunological heterogeneity of tumors. *Nature*, **280**, 358.

OWEN, J. J. T. & SEEGER, R. C. (1973) Immunity to tumours of the murine leukaemia–sarcoma virus complex. *Br. J. Cancer*, **28**, Suppl. I, 26.

POSTE, G. & FIDLER, I. J. (1980) The pathogenesis of cancer metastasis. *Nature*, **283**, 139.

RUSSELL, S. W., GILLESPIE, G. Y., HANSEN, C. G. & COCHRANE, C. G. (1976) Inflammatory cells in solid murine neoplasms. II. Cell types found throughout the course of Moloney sarcoma regression or progression. *Int. J. Cancer*, **18**, 331.